# LawRec: Automatic Recommendation of Legal Provisions Based on Legal Text Analysis

**DOI:** 10.1155/2022/6313161

**Published:** 2022-09-14

**Authors:** Min Zheng, Bo Liu, Le Sun

**Affiliations:** ^1^Hubei University of Science and Technology, Xianning, China; ^2^Nanjing University of Information Science and Technology, Nanjing, China

## Abstract

Smart court technologies are making full use of modern science to promote the modernization of the trial system and trial capabilities, for example, artificial intelligence, Internet of things, and cloud computing. The smart court technologies can improve the efficiency of case handling and achieving convenience for the people. Article recommendation is an important part of intelligent trial. For ordinary people without legal background, the traditional information retrieval system that searches laws and regulations based on keywords is not applicable because they do not have the ability to extract professional legal vocabulary from complex case processes. This paper proposes a law recommendation framework, called LawRec, based on Bidirectional Encoder Representation from Transformers (BERT) and Skip-Recurrent Neural Network (Skip-RNN) models. It intends to integrate the knowledge of legal provisions with the case description and uses the BERT model to learn the case description text and legal knowledge, respectively. At last, laws and regulations for cases can be recommended. Experiment results show that the proposed LawRec can achieve better performance than state-of-the-art methods.

## 1. Introduction

Artificial intelligence technology has flourished in both academy and industry. Face recognition, voice recognition, and other intelligence technologies are developing rapidly [1]. Intelligent products such as smart speakers and sweeping robots have entered thousands of households. Smart court technologies are making full use of modern science such as the artificial intelligence, Internet of things, big data, and cloud computing to promote the modernization of the trial system and trial capabilities, thereby improving the efficiency of case handling and achieving convenience for the people [2].

With the step-by-step advancement of the court's informatization process, the record carrier of case information and adjudication process has been transformed from paper to electronic filing [3]. Relying on the rapid development of the Internet, case records are not limited to a certain court, city, or province, but they have a nationwide network of judgment documents. These conditions have led to the creation of a huge library of judicial documents with standardized formats. The judgment document is the record and summary of the case, the facts of the case, the trial process, and the basis of the trial after the judge completes the trial [4]. It contains a large amount of data information. These accumulated judgment documents have become a powerful data support for legal research, providing a good data foundation for subsequent intelligence.

Article recommendation is an important part of intelligent trial. Because the law is the basis for the outcome of the trial, the judge must handle the case in accordance with the law. Therefore, the statutes represent the direction of the trial of the case to a certain extent. In addition, the value of legal recommendations is also reflected in the help they can provide to various roles involved in legal cases [5]. For judges trying cases, if they can learn from the trial information of similar cases in the past, they can handle cases more efficiently. For lawyers who defend the plaintiff and the defendant, if they can quickly find the applicable laws and regulations from a variety of laws and regulations, they can better defend their clients with stronger arguments. For plaintiffs and defendants who lack legal knowledge, without the help of professionals, they have no way of knowing whether there are suitable statutes to protect their rights and interests, and a system that can correctly predict statutes can help them save time and money in legal consultation [6].

For ordinary people without legal background, the traditional information retrieval system that searches laws and regulations based on keywords is not applicable because they do not have the ability to extract professional legal vocabulary from complex case processes [7]. The Bidirectional Encoder Representation from Transformers (BERT) model has powerful text representation and text understanding capabilities. This model has been widely used in semantic understanding-based fields, such as entity recognition, text classification, and other fields, but it is rarely used in the field of legal recommendation. This paper proposes a law recommendation framework, called LawRec based on BERT and Skip-Recurrent Neural Network (Skip-RNN) models [8], which intends to integrate the knowledge of legal provisions with the case description and uses the BERT model to learn the case description text and legal knowledge, respectively. At last, laws and regulations for cases can be recommended.

The paper structure is as follows: Section 2 introduced the related work of law recommendation.  Section 3 describes the LawRec framework.  Section 4 gives the experiment analysis and results.  Section 5 makes a summarization.

## 2. Related Work

At present, the research on judicial intelligence at home and abroad has achieved certain results. Work [9] constructed a dataset of 2.6 million criminal cases for trial prediction, including case facts as input and three predictors of citations, charges, and jail time. Reference [10] proposed a model for predicting whether a court will uphold or overturn a judgment. By analyzing the lawyer's historical case handling and court trial performance, the lawyer is scored, and then the lawyer is recommended according to the current case type. There are also studies conducted on criminal cases. Work [11] used a bidirectional Gated Recurrent Unit (GRU) model to predict criminal charges based on court-finding facts and legal grounds. Work [12] extracted logical basis from case facts through reinforcement learning, which enhanced the interpretability of crime prediction. Work [13] regarded the court opinion as the interpretation of the crime and used the conditional seq2seq model to generate the judge's judgment analysis process according to the criminal facts of the criminal case.

In terms of recommendation algorithms, recommender systems first appeared in the 1990s [14, 15], which provide users with suggestions through historical information analysis and help users quickly find useful information. Collaborative filtering [16] is one of the most widely used algorithms in the field of recommender systems, involving social, shopping, finance, law, and other fields. Work [17] proposed a collaborative filtering-based network news system to help people find favorite articles in a large stream of information. Work [18] proposed content-based collaborative filtering to solve the problem that the workload of traditional methods increases with the increase of system participants.

Considering the recommendations of the law, some scholars have conducted part of the research. Most of them are aimed at expert users such as judges and lawyers and focus on information retrieval or keyword-based classification. How to make computers understand the meaning of natural language correctly has always been a topic of academic research. In recent years, the research of neural network algorithm has made breakthrough progress in this area. Work [19] applied neural network to lexical error correction. Work [20] proposed a neural network combining dynamic pooling and recurrent autoencoders for paraphrase detection. Work [21] used CNN for text classification and achieved better results than other models. Work [22] designed a court judgment evaluation model. The evaluation model is based on BP neural network. Work [23] designed a model based on bidirectional long short-term memory networks. The model can recognize legal text.

## 3. LawRec: BERT-Based Law Recommendation Framework

The Bidirectional Encoder Representation from Transformers (BERT) model has powerful text representation and text understanding capabilities. This model has been widely used in semantic understanding-based named entity recognition, text classification, and other fields, but it is rarely used in the field of legal recommendation. This paper proposes a law recommendation framework, called LawRec, based on BERT and Skip-RNN models, which intends to integrate the knowledge of legal provisions with the case description and uses the BERT model to learn the case description text and legal knowledge, respectively. At last, laws and regulations for cases can be recommended.

**Figure 1:**
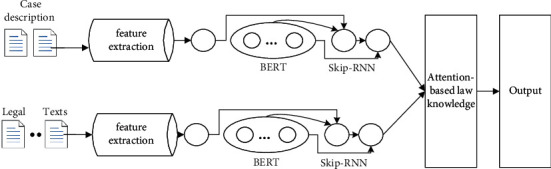
Framework of LawRec based on BERT and Skip-RNN.

This paper proposes a law recommendation method based on knowledge fusion in the field of judicial law. The overall structure is shown in Figure 1. The proposed model includes rule extraction of laws, BERT training, and rule recommendation of laws. The legal knowledge extraction layer extracts keywords from the legal knowledge in the judicial field to obtain the legal knowledge. The BERT model performs semantic representation of the case description text and legal knowledge based on the Skip-RNN. Therefore, the semantic representation vector can be obtained. The legal rule knowledge integration layer is mainly based on the attention mechanism. The legal rule knowledge integration layer can realize the feature fusion of legal rule knowledge features and case description. And, the case description feature vector fused with legal rule knowledge can be obtained. The legal recommendation layer is like the traditional legal recommendation framework and adopts the idea of text classification to achieve the final legal recommendation.

### 3.1. Feature Extraction

The legal provisions for specific types of cases are generally long. To accurately locate the core knowledge of legal provisions, this paper extracts the keywords of legal provisions and finally obtains the core knowledge of legal provisions, which is convenient for subsequent follow-up.

### 3.2. BERT Model for Feature Modelling

For the text description *E*=[*E*_1_, *E*_2_,…, *E*_*n*_] and legal knowledge *L*=[*L*_1_, *L*_2_,…, *L*_*m*_] of a specific case, where *n* represents the length of the case description text, *m* is the length of the legal text knowledge, this paper uses the BERT model to characterize them, respectively. Based on the BERT model, we get the specific text description vector which is as follows:

(1) $$\begin{array}{c} \begin{array}{c} G_{E}=BERT\left(E\right)\\ G_{L}=BERT\left(L\right) \end{array}\text{,} \end{array}$$

where *G*_*E*_ and *G*_*L*_ represent the BERT-based case text description vector and legal rule knowledge representation vector, respectively. To improve the continuous representation ability of text sequence information, a Skip-RNN layer is added after the BERT pretraining module. For longer sequences, Skip-RNN adds a skip gate, which outputs the number of steps to be jumped according to the current state, thereby speeding up the training. Skip-RNN can learn forward and backward information, improve the contextual and contextual feature information extraction capabilities of text feature vectors, and solve long-distance dependencies. Specifically,

(2) $$\begin{array}{c} \begin{array}{c} G_{E}=SkipRNN\left(G_{E},G_{E1}\right)\\ G_{L}=SkipRNN\left(G_{L},G_{L1}\right) \end{array}\text{.} \end{array}$$

Among them, *G*_*E*_ and *G*_*E*1_ are the forward and backward outputs of the case description text hidden layer, respectively, and *G*_*L*_ and *G*_*L*1_ are the forward and backward outputs of the legal knowledge hidden layer, respectively.

### 3.3. Legal Knowledge Representation

To enhance the importance of legal article knowledge, attention mechanism is usually used to fuse legal article knowledge and case description. Finally, a case description that integrates legal knowledge can be obtained. The attention calculation formula of case description feature *G*_*E*_ and legal knowledge feature *G*_*L*_ is as follows:

(3) $$\begin{array}{c} f\left(G_{E}\left(i\right),G_{L}\right)=G_{E}\left(i\right)G_{L}^{t}\text{,} \end{array}$$

where *G*_*E*_(*i*) represents the feature of the *i*-th text described by the text. Then, normalize the knowledge features of legal articles and the feature attention of each case text, and the specific formula is as follows:

(4) $$\begin{array}{c} t_{k}=softmax\left[f\left(G_{E}\left(k\right),G_{L}\right)\right]\text{,} \end{array}$$

where *t*_*k*_ represents the attention vector of the *k*-th text describing the knowledge features of external legal articles and the facts of the case.

Finally, the case description text features are weighted and summed based on the attention weight to obtain the case description vector *G*_*EL*_ fused with legal knowledge. The attention mechanism can focus on useful information and ignore unimportant information. The principle of this mechanism is to calculate the weight corresponding to the information. The greater the weight, the more important the information is.

(5) $$\begin{array}{c} G_{EL}=\sum t_{i}G_{E}\left(i\right)\text{.} \end{array}$$

### 3.4. Law Recommendation

Like previous legal recommendation methods, the prediction process is still divided into three steps: (1) describe the fusion case features and legal knowledge, (2) perform linear transformation, and (3) use softmax to achieve prediction. *g*_1_ is the prediction result:

(6) $$\begin{array}{c} g_{1}=softmax\left(G_{el}\right)\text{.} \end{array}$$

This paper uses cross-entropy loss to minimize the prediction error between the output result and the label. The cross-entropy loss formula is as follows:

(7) $$\begin{array}{c} loss=-\sum_{i}l_{1}\log l_{n}+\lambda {\left\|\theta \right\|}^{2}\text{,} \end{array}$$

where *l*_n_ is the label vector predicted by the model in this paper, *l*_1_ is the labeled normal label vector, and *l*_2_ is the regularization term.

## 4. Experimental Results and Analysis

### 4.1. Experimental Dataset

In the Fayan Cup dataset, an experiment was conducted on the legal article recommendation task [24]. In order to achieve a relatively balanced dataset, this paper deleted some low-frequency legal articles in the Fayan Cup dataset and deleted some invalid samples and stop words, and finally, the training set selected in this paper is 800,000, and the validation set and test set are each 50,000. The number of law labels selected in this paper is about 1.1 million. The scale of the data set is shown in Table 1.

Since this article uses the fact description and the law part of the data, the law recommendation data includes the case fact description text and the specific law label. The specific form of the data is shown in Table 2.

Like the traditional law recommendation task, this paper uses the *F*_1_ value as the evaluation index:

(8) $$\begin{array}{c} F_{1}=\frac{2\text{PR}}{P+R}\text{,} \end{array}$$

where *P* is the precision rate and *R* is the recall rate.

### 4.2. Model Construction and Experimental Parameter Settings

The model used in this paper is built with PyTorch, and the specific parameters are designed as follows: the word vector dimension is 320, the number of Skip-RNN hidden layer units is 440, the learning rate is 0.002, the dropout is set to 0.6 to prevent overfitting, and the batch size is 64.

### 4.3. Comparative Model and Analysis of Experimental Results

To prove the effectiveness of the method proposed in this paper, we compare and analyze the three aspects of traditional law recommendation method, law knowledge ablation, and BERT pretraining model ablation.

- Transformer [25]: it has achieved very good results in the field of machine translation.
- SVM [26]: it was first used to solve the two-classification problem in pattern recognition, and it has achieved good classification results in the fields of text classification, handwriting recognition, and image processing.
- TextRnn [27]: it is a model that uses RNN for text classification.
- FastText [28]: its biggest feature is that the model is simple, the training speed is very fast, and it is widely used in the field of text classification.
- BERT [29]: it has strong text representation ability and achieves good results in various tasks of deep learning.
- Text CNN [30]: it is a typical model using CNN for text classification.

The specific experimental results are shown in Table 3.

Experimental results show that the LawRec based on BERT significantly outperforms traditional data-driven methods in terms of the accuracy rate *P*, recall rate *R*, and *F*_1_ values.

In order to verify the impact of the BERT pretraining model on the experimental performance, this paper uses the BERT pretraining model and the word2vec representation model to conduct a comparison experiment on the legal recommendation task in the Fayan Cup public test data set. The specific experimental results are shown in Table 4.

It can be seen from Table 4 that the model based on BERT characterization can significantly improve the *F*_1_ result of legal recommendation. This is because the BERT pretraining model has strong representation ability for legal knowledge and case description text, so it improves the legal recommendation's performance.

In order to verify the impact of incorporating legal knowledge on the performance of legal recommendation, this paper conducts a comparative experiment of incorporating legal knowledge ablation, and the specific experimental results are shown in Table 5.

It can be seen from Table 5 that adding rule knowledge can greatly improve the *F*1 value of the testing set. This is because the fusion of legal article knowledge can improve the feature extraction performance of case text description to a certain extent, so that the extracted case text features are more inclined to legal article knowledge, so the performance of legal article recommendation is improved. The experimental results demonstrate the effectiveness of incorporating external knowledge of legal articles on the legal article recommendation task.

To further illustrate that the law recommendation model incorporating legal knowledge can effectively solve the problem of recommending confusing laws, here is a specific analysis based on Article 252 of the Criminal Law (crime of intentional injury) and Article 252 of the Criminal Law (crime of intentional homicide).  Table 6 is a case of intentional injury that was mispredicted as intentional homicide in a model that did not incorporate legal knowledge. From the description of the case, we can see that there are a large number of keywords that distinguish the crime of intentional injury from the crime of intentional homicide, such as intentional injury, body, negligent death, cruel means, serious injury, serious disability, and other keywords in the crime of intentional injury. Therefore, we conclude that the legal recommendation model incorporating legal knowledge can accurately distinguish the crime of intentional injury and the crime of intentional homicide. This example shows to a certain extent that the legal recommendation model incorporating legal knowledge can effectively solve the problem of confusing legal recommendations.

To more intuitively illustrate the process of legal recommendation, an example analysis of fraud crime is given, as shown in Tables 6 and 7.

Based on the combination of keywords (e.g., “public and private property” and “large amount of money”) and the attention mechanism, the attention of fraud crimes can be increased. This paper combines legal knowledge and case description text to achieve targeted feature extraction, thereby achieving accurate legal recommendation.

## 5. Conclusion

The traditional information retrieval system based on keyword search for laws and regulations is not suitable for ordinary people without professional legal knowledge. Therefore, it is necessary to propose a legal recommendation framework to help them extract professional legal vocabulary from complex case processes. This paper proposed a law recommendation framework, called LawRec, based on BERT and Skip-RNN models, which intends to integrate the knowledge of legal provisions with the case description and uses the BERT model to learn the case description text and legal knowledge, respectively. At last, laws and regulations for cases can be recommended. Experiment results show that the proposed LawRec can achieve better performance than state-of-the-art methods. The accuracy of LawRec is 92%, which is 12% higher than that of the model that does not incorporate legal knowledge.

## Figures and Tables

**Table 1:** Data set size.

Training set/number	Validation set/number	Test set/number
800,000	50,000	50,000

**Table 2:** Case description legal data form.

Description of the facts: “After the trial, it was found that at about 20:00 on June 22, 2020, the defendant Zheng Lily, together with Wang and Liu (2 persons were sentenced), drove a red van to the ×× county ×× township × village ×× expressway four-standard project department. There were 60 anchorages on the ×× construction site. During the escape, the project manager Shao found out that Zhang and Zeng got out of the car and threatened Shao, Zhang injured Shao, and the defendant Zhen did not get out of the car or threaten the victim during the process. The last three people drove away from the scene. After identification, the value of the stolen items was 4562 yuan. The above facts, the defendant Liu has no objection during the trial, and there is the confession of the defendant Sun in the public security organs, the testimony of the witnesses Ali, Lily, Wang, Perter, and others, and the appraisal report of the assets involved in the case, criminal judgment, and the evidence of the defendant Liu arrival at the case and his household registration information are sufficient to confirm.”

**Table 3:** Comparative experimental results.

Data set	Model	*P*	*R*	*F*_1_	Test set	Model	*P*	*R*	*F*_1_
Test	Transformer	0.77	0.76	0.76	Test	Transformer	0.75	0.74	0.74
	SVM	0.84	0.83	0.83		SVM	0.85	0.84	0.84
	TextRnn	0.83	0.82	0.82		TextRnn	0.81	0.80	0.80
	TextRnn	0.81	0.80	0.80		TextRnn	0.82	0.81	0.81
	FastText	0.85	0.84	0.84		FastText	0.85	0.83	0.83
	BERT	0.82	0.80	0.81		BERT	0.82	0.80	0.81
	Text CNN	0.85	0.84	0.84		Text CNN	0.86	0.85	0.85
	LawRec	0.92	0.91	0.91		LawRec	0.93	0.90	0.90

**Table 4:** Comparison experiment between BERT model and traditional representation model.

Data	Model	*F*_1_
Test set	Traditional model	0.82
Test set	BERT model	0.91

**Table 5:** Comparative experimental results of external legal knowledge ablation.

Data	Model	*F*_1_
Test set	Models that incorporate legal knowledge	0.92
Test set	Models that do not incorporate legal knowledge	0.80

**Table 6:** Cases of intentional injury.

Description of the facts: “The XX city procuratorate charged that on the afternoon of July 20, 2020, the defendant, Zhang, learned from his daughter, Zhang, that the victim, Liu, wanted to trouble Liu. Later, Zhang and Liu talked on the phone many times, and the two scolded each other on the phone and agreed to meet at the west bridge in XX town, XX city. At about 12:00 on the same day, Zhang drove Liu and Dan to the west bridge, after which Zhang fought with the victims Liu, Peter, and Lily. During the tussle, Wang took out a crowbar from the trunk of his car and used the crowbar to injure Lily's right thumb and Liu's head and body. It was identified that Lily's injury was a second-level serious injury, and the injury suffered by Peter was minor.”

Intentional injury crime: whoever intentionally injures another person's body shall be sentenced to fixed-term imprisonment of not more than three years, criminal detention or public surveillance. Who intentionally murders shall be sentenced to life imprisonment, death, or fixed-term imprisonment of not less than 20 years? If the circumstances are relatively minor, it shall be sentenced to fixed-term imprisonment of not less than 2 years but not more than 20 years.

Intentional homicide: who intentionally murders shall be sentenced to life imprisonment, death, or fixed-term imprisonment of not less than 20 years. If the circumstances are relatively minor, it shall be sentenced to fixed-term imprisonment of not less than 2 years but not more than 20 years.

**Table 7:** Case analysis on the crime of fraud.

Description of the facts: “The public prosecution alleges that from April 2015 to March 2017, the defendant Ju fabricated the facts of handling work, in the name of the cost of handling the work, successively defrauded Li of RMB 800,000, Peter of USD 35,000, Yang of USD 12,000, Yu of USD 24,000, and Ju of USD 37,000; fraudulently obtained USD 240,000 from Zhang in the name of the cost of handling work, purchasing a house, and treating illnesses by fabricating facts such as working, purchasing a house, and being ill. Defendant Peter also tried to defraud Liu of USD 22,000 by fabricating the fact of handling work and in the name of the cost of handling work.”

Fraud crime: refers to the act of defrauding a large amount of public and private property by using fictitious facts or concealing the truth for the purpose of illegal ownership.

## Data Availability

The labeled datasets used to support the findings of this study are available from the corresponding author upon request.
